# Trends in obstructive sleep apnea disease severity over nearly two decades: update on the VA San Diego experience

**DOI:** 10.1093/sleepadvances/zpae036

**Published:** 2024-06-07

**Authors:** Brandon Nokes, Tania Zamora, Yzabel Velazquez, Shah Golshan, Cesar Cervantes-Gomeros, Will Perrine, Robert Barker, Atul Malhotra, Kathleen F Sarmiento, Carl Stepnowsky

**Affiliations:** Sleep Medicine Section, Department of Medicine, VA San Diego Healthcare System, San Diego, CA, USA; Department of Medicine, Division of Pulmonary, Critical Care, Sleep Medicine and Physiology, University of California at San Diego, La Jolla, CA, USA; Health Services Research & Development Unit, VA San Diego Healthcare System, San Diego, CA, USA; Health Services Research & Development Unit, VA San Diego Healthcare System, San Diego, CA, USA; Health Services Research & Development Unit, VA San Diego Healthcare System, San Diego, CA, USA; Sleep Medicine Section, Department of Medicine, VA San Diego Healthcare System, San Diego, CA, USA; Sleep Medicine Section, Department of Medicine, VA San Diego Healthcare System, San Diego, CA, USA; Sleep Medicine Section, Department of Medicine, VA San Diego Healthcare System, San Diego, CA, USA; Department of Medicine, Division of Pulmonary, Critical Care, Sleep Medicine and Physiology, University of California at San Diego, La Jolla, CA, USA; Sleep Medicine Section, Department of Medicine, VA San Francisco Healthcare System, San Francisco, CA, USA; Health Services Research & Development Unit, VA San Diego Healthcare System, San Diego, CA, USA; Department of Medicine, University of California at San Diego, La Jolla, CA, USA

## Abstract

**Study Objectives:**

The Sleep Program at the VA San Diego Healthcare System (VASDHS) started a patient database over twenty years ago for its home sleep apnea testing (HSAT) program. An analysis of ten years of diagnostic HSAT data was reported on over 12 500 patients in 2014. Over this time period, severe obstructive sleep apnea (OSA) decreased in frequency. In contrast, mild OSA increased in frequency and was the most frequently reported severity in our analysis. In more recent times, the 2021 continuous positive airway pressure (CPAP) crisis created difficulties in dispersing CPAP therapies to individuals including Veterans with OSA, prompting our group to reexamine the HSAT database.

**Methods:**

A retrospective review was performed of the local clinical database of HSAT diagnostic testing of 8,928 sleep studies from 2018 to 2022.

**Results:**

The overall mean apnea–hypopnea index (AHI) decreased from 40.4/hour (2004) to 24.3/hour (2022) (*p* < .001). The two time periods were examined separately. For 2004–2013, it was found that the mean AHI in 2004 was not significantly different from the mean AHI in 2005, 2006, or 2007 but was significantly different from the mean AHI in each year from 2008 (mean AHI = 30.7/h) to 2013 (mean AHI = 26.1/hour). For 2019–2022, the mean AHI did not significantly differ between the 4 years.

**Conclusions:**

These findings have implications for OSA therapies. Additionally, the high prevalence of mild sleep apnea, which is typically associated with lesser adherence to PAP therapy, further highlights the importance of non-PAP alternatives to improve treatment effectiveness.

**Statement of Significance**

This is a retrospective review of home sleep testing outcomes within a large sleep testing center at the San Diego VA. These findings demonstrate that although obstructive sleep apnea is common, most cases are mild in severity. In consideration of the 2021 continuous positive airway pressure crisis, these data might be leveraged for therapeutic planning should a similar CPAP shortage recur.

The Veterans Health Administration (VA) is one of the largest integrated healthcare systems in the United States. Obstructive sleep apnea (OSA) is common within VA. Key risk factors for OSA include aging, weight, and male gender, which are highly prevalent in the VA population. The Sleep Program at the VA San Diego Healthcare System (VASDHS) started a patient database over twenty years ago for its home sleep apnea testing (HSAT) program. An analysis of 10 years of diagnostic HSAT data was reported on over 12 500 patients in 2014 [1]. Over this time period, severe OSA decreased in frequency. In contrast, mild OSA increased in frequency and was the most frequently reported severity in our analysis [1].

In more recent times, the 2021 global safety recall of Philips positive airway pressure (PAP) devices and ensuing COVID-19-related supply chain crisis led to difficulties in the ability to prescribe PAP therapies to individuals [2] including Veterans with OSA [3]. Our VA and others trialed a number of PAP alternatives within this context [4]. The PAP crisis also prompted VASDHS to reexamine the prevalence and severity of OSA to improve our understanding of the population we serve and to facilitate forecasting and planning for therapeutic options. This analysis focuses on an update on the last four years, 2019–2022, in comparison to 2004–2013.

## Materials and Methods

A retrospective review of the local clinical database of HSAT diagnostic testing (Nox T3, Nox Medical, Reykjavik, Iceland) was conducted. The database contained 8928 sleep studies from 2018 to 2022. Invalid studies were removed. Only initial diagnostic studies were included. Efficacy studies were presumed to be the second HSAT performed on the same individual patient and were excluded. Home sleep studies were scored with 3% desaturation criteria for hypopneas and apnea–hypopnea index (AHI)3% is reported for all time periods [5]. For the purpose of this study, AHI is synonymous with the respiratory event index and respiratory disturbance index. Negative studies were defined as an AHI of less than 5 events/h, mild between 5.0 and 14.9 events/h, moderate between 15 and 29.9 events/h, and severe greater than or equal to 30 events/h. Severity classification definitions did not change during the study time periods. Demographic data were not available.

## Results

The overall mean AHI decreased from 40.4/h (2004) to 24.3/h (2022; *p* < .001). The two time periods were examined separately. For 2004–2013, it was found that the mean AHI in 2004 was not significantly different from the mean AHI in 2005, 2006, or 2007 but was significantly different from the mean AHI in each year from 2008 (mean AHI = 30.7/h) to 2013 (mean AHI = 26.1/h). For 2019-2022, the mean AHI did not significantly differ between the 4 years.

### Period 1 (2004–2013)

Of 11 099 studies were positive for OSA during this period. The distribution of OSA severity changed during this time, such that severe OSA decreased by half (from 60% to 30%), mild OSA increased seven-fold (from 5% to 35%), and moderate OSA remained stable.

### Period 2 (2019–2022):

**Figure 1.**
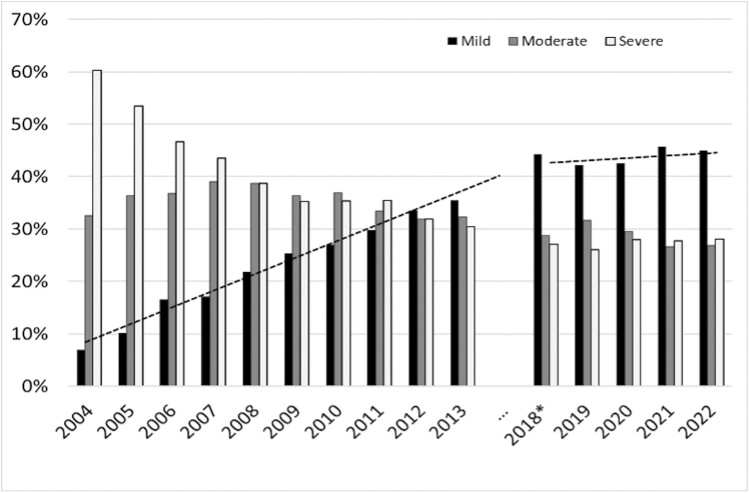
OSA severity from 2004 to 2022. The chart shows increasing rates of mild OSA from 2004 to 2013, and stable rates of mild OSA from 2018 to 2022. The trend line is for mild OSA. * Note: 2018 data only included the last four months of the year.

Of the 8928 diagnostic studies, 2564 were negative studies (28.7%). Of the 6364 positive studies, 43.3% were mild, 28.8% were moderate, and 27.9% were severe. The distribution of OSA severity levels were stable across these years (mild: 42%–45%; moderate: 27%–32%; and severe: 26%–29%). The mean AHI did not significantly change from 2018 (24.1/h ± 20.6) to 2022 (25.1/h ± 21.2) (overall mean 24.4/h ± 20.3; range: 5–140; NS). The mean BMI was 30.6 kg/m^2 (11.7). The distribution of OSA severity within our center over time can be seen in Figure 1. The number of HSATs per year was similar between 2013 and 2022.

## Discussion

Based on this extensive retrospective review of diagnostic testing at a single, high-volume HSAT program, it appears that trends in OSA severity have stabilized over the past 5 years, with mild OSA accounting for the highest percentage (43%), followed by moderate (29%) and severe (28%). Since tracking of sleep study results started in 2004, a significant decrease in severe cases was observed (60% to 30%) with a corresponding increase in mild OSA (7% to 43%). These findings parallel a 2019 MedXcloud analysis, which suggested that up to 1 billion individuals globally have OSA, with mild OSA comprising ~55% of all OSA cases [6]. Notably, there was a recent study from a single lab with polysomnography-based sleep apnea trends reporting between 1989 and 2013, where they noted an increase in OSA severity as well as an increase in age, female patients with OSA, and BMI. However, this cohort of patients differs significantly from ours based on the examined use of multiple diagnostic sleep technologies, transitions in scoring and interpretation methods, and is not directly comparable [7].

Notably, since our initial analysis, the number of Veterans tested for OSA using predominantly HSAT systems has markedly increased due to the focused efforts of VA to improve access to sleep testing services [8, 9]. With marked innovation occurring in treatment options, severity of sleep apnea now has significant implications on which therapies are prescribed. Identifying patient-centered treatment options for these patients is thus an important priority. We speculate that the trends in OSA severity over time may also be in part related to an increase in community awareness regarding OSA and greater recognition of the interconnectedness of OSA with traumatic brain injury and mood and stress disorders.

Our study has several limitations in that it is a retrospective review of single-night data from an HSAT system, may under-represent the AHI [10]. Moreover, the lack of demographic, socioeconomic, and data on comorbidities across both study periods limits the interpretation of our findings. It is also unknown how the evolution of HSAT quality over time influenced the sensitivity of AHI event detection, but this is unlikely to explain the magnitude of difference in our findings. We suspect the increased number of mild studies reflects changing patterns of referrals as awareness of sleep apnea intersecting with other medical conditions increased during the study period. In other words, more patients were referred for sleep testing from mental health clinics (for post-traumatic stress disorder, depression, traumatic brain injury), as well as from cardiology (for arrhythmias), urology (for nocturia), and ophthalmology (for glaucoma and floppy eyelid syndrome). Referrals also increased for testing referrals as part of disability evaluations for the Veterans Benefits Administration. Lastly, there are insufficient granular data to explain why each of these studies was ordered and despite attempts to exclude them, some studies may have been efficacy studies while on continuous positive airway pressure, while using an oral appliance, or after behavioral interventions such as weight loss related to diet and exercise.

Nonetheless, these findings offer important insights into OSA burden and severity within a large VA Medical Center. The shifts in severity captured in the current study likely reflect increased OSA awareness and changes in referral patterns, acknowledging the importance of healthy sleep in a broad range of medical and mental health disorders. These findings have implications for system-level planning regarding therapies used to treat OSA. Additionally, the high prevalence of mild sleep apnea, which is typically associated with lesser adherence to PAP therapy, further highlights the importance of non-PAP alternatives to improve treatment effectiveness [11]. Despite ongoing debates as to whether mild OSA should be treated, the current consensus is that quality of life and symptoms may improve in symptomatic individuals [12]. In conclusion, this study demonstrates stability in OSA severity trends among Veterans over the past five years and highlights the opportunity VA has to further individualize treatment options for Veterans based on these results, particularly for those with mild OSA.
